# Sex differences in social and emotional insight in youth with and without autism

**DOI:** 10.1186/s13229-023-00541-w

**Published:** 2023-03-04

**Authors:** Hunter Mattern, Meredith Cola, Kimberly G. Tena, Azia Knox, Alison Russell, Maggie Rose Pelella, Aili Hauptmann, Maxine Covello, Julia Parish-Morris, Joseph P. McCleery

**Affiliations:** 1 Department of Psychology & Kinney Center for Autism Education and Support, Saint Joseph’s University, Philadelphia, USA; 2 Center for Autism Research, Children’s Hospital of Philadelphia, Philadelphia, USA; 3 Department of Psychology, Penn State University, 140 Moore Building, University Park, PA 16802 USA; 4 Perelman School of Medicine, University of Pennsylvania, Philadelphia, USA

**Keywords:** ASD, Gender differences, Girls, Women, Social understanding, Emotional knowledge

## Abstract

Autism was formally recognized by the medical community in the first half of the twentieth century. Almost 100 years later, a small but growing literature has reported sex differences in the behavioral expression of autism. Recent research has also begun to explore the internal experiences of individuals with autism, including social and emotional *insight*. The current study examines sex differences in language-based markers of social and emotional insight in girls and boys with autism and non-autistic peers during semi-structured clinical interviews. Sixty-four participants aged 5 to 17 years were individually matched on chronological age and full-scale IQ to form four groups: autistic girls, autistic boys, non-autistic girls, and non-autistic boys. Transcribed interviews were scored using four scales that index aspects of social and emotional insight. Results revealed the main effects of diagnosis, such that youth with autism exhibited lower insight than non-autistic youth on scales indexing social cognition and object relations, emotional investment, and social causality. With regards to sex differences, across diagnoses, girls were rated higher than boys on the social cognition and object relations, emotional investment, and social causality scales. Examined within each diagnosis separately, clear sex differences emerged: both autistic and non-autistic girls demonstrated better social cognition and understanding of social causality than boys in their respective diagnostic groups. No within-diagnosis sex differences were found on the emotional insight scales, however. These results suggest that relatively enhanced social cognition and understanding of social causality in girls may be a population-level sex difference that is preserved in autism, despite the core social challenges that characterize this condition. The current findings reveal critical new information about insight into social and emotional thinking and relationships in autistic girls versus boys that have important implications for improving identification and designing effective interventions.

**Supplementary Information:**

The online version contains supplementary material available at 10.1186/s13229-023-00541-w.

## Background

Autism is a neurodevelopmental condition that affects 1 in 44 US school children [1], and is characterized by heterogeneous profiles of social communication difficulties and restricted and repetitive patterns of behaviors and interests [2]. Although it is widely accepted that boys are 4 × more likely to be diagnosed with autism than girls [3], there is emerging evidence to suggest that a significant proportion of autistic girls are missed or misdiagnosed in childhood, only to be later diagnosed with the condition as adults [4]. Unfortunately, this means that many autistic girls miss key opportunities to access early intervention and support. Autistic girls are not necessarily undiagnosed in childhood because their autism requires less support; in fact, research shows that even when girls have comparable condition profiles to boys, they are still diagnosed with autism less often than their male counterparts [5]. One possible explanation for this finding is that autism manifests *differently* in girls and boys, and thus may not be as obvious to clinicians looking for the male-standard prototype of what “seems autistic.” In other words, just as girls and boys face different social demands, girls may express their autistic symptoms in ways that are distinct from male patterns.

Differences in the behavioral presentation of autism, including how children talk about social and emotional topics, could contribute to persistent issues associated with identifying and supporting autistic girls. A growing body of research has shown that autistic girls’ and boys’ behavior differs across multiple domains including social attention [6], gesture [7], imaginative play [8], friendships [9, 10], social motivation [11], social reciprocity, and language [12–18]. However, potential sex differences in subtle “hidden” dimensions like social and emotional insight are not yet well understood. In fact, it is possible that common misconceptions about social and emotional insight in autism (e.g., that it is completely absent, or unimportant, and thus has not been studied) could make it more difficult to identify certain subgroups that demonstrate greater than expected levels of insight. It is important to note, that these differences in social and emotional insight between autistic boys and girls have been primarily observed in those with high intellectual and verbal abilities. The social and emotional understanding of autistic boys and girls with intellectual disabilities and limited language warrant exploration in future research.

### Social and emotional insight in autism

Social communication challenges are a core component of the autism diagnostic criteria [2]. These social communication difficulties can manifest differently across individuals and often include challenges with social and emotional understanding and insight that translate into observable “gaffes” and repeated failure to meet social goals. Emotional insight refers to the understanding of emotions, the ability to verbally explain how an individual feels both mentally and physically when experiencing a specific emotion, the ability to recall past experiences that evoked a certain emotion, the impact an individual’s emotions and associated actions can have on others, and the ability to describe when people in the individual’s life may experience a certain emotion [19]. Notably, the majority of previous research on emotional understanding in autism focused on improving individuals’ identification of emotions by training them to identify facial emotions from static pictures [20–22]. Although this research provided important information about participants’ ability to comprehend basic emotions in faces, these results cannot speak to the individuals’ recognition or knowledge of more complex emotions or internal states that are not clearly presented through facial expressions. This is a critical consideration, as the social–emotional challenges faced by autistic individuals in day-to-day life are more complex than static pictures; they are embedded within a rich, dynamic social context.

Prior research assessing autistic participants’ abilities to describe what an emotion is or to provide examples of emotions from their own experiences has been limited. However, evidence to date suggests that autistic individuals are typically able to describe and provide examples of basic emotions, such as happy, sad, and mad; but have more difficulty describing complex emotions compared to a non-autistic control group [23].

### Sex differences in social and emotional insight

A large body of prior research shows that non-autistic girls exhibit greater social understanding, skills, and insight than boy peers, in both verbal and nonverbal domains [9, 24, 25]. However, it remains unknown whether population-level sex differences in social–emotional insight—namely deep internal understanding of social dynamics and emotional experiences—are preserved in girls and boys with autism.

Behaviorally, some studies show that autistic girls and women appear more socially or emotionally competent than they actually are [26, 27]. For example, teachers have reported substantially fewer concerns about social skills in school-aged autistic girls compared to boys, in part because girls “blend in” or “camouflage” with peers at the surface level of observed behavior [28] despite internal struggles [29, 30]. In studies that recorded behaviors of children during recess, autistic girls were typically seen in groups and spent most of their free time socializing, whereas autistic boys spent more time in structured activities or were more isolated [31].

Girls were observed to spend more time in social activities in which they experienced more social situations and, in turn, had increased opportunities to learn and develop social insight, compared to autistic boys. Compared with boys, autistic girls are more likely to be accepted by non-autistic girls as fringe members of female social groups until adolescence, when friendships among girls evolve and begin to require considerably more nuanced social skills [32, 33]. Given that young girls diagnosed with autism have been observed to exhibit somewhat higher levels of social abilities compared to boys, they may rely upon this increased social understanding and insight in order to camouflage or otherwise work their way through social situations. While it is distinctly possible that girls with autism have higher levels of internal social–emotional insight compared with boys with autism, the potential elevation in insight has not yet been directly examined.

Sex refers to the biological characteristics that define humans as female or male. While these biological characteristics tend to differentiate humans as females and males, they are not entirely mutually exclusive and there are individuals who possess both. Gender refers to the characteristics of girls, boys, women, and men that are socially constructed. That is, gender relates to norms, behaviors, and roles associated with being a girl, boy, woman, or man. As a social construct, gender varies from society to society and can change over time and with social context, both within an individual and across individuals.

### Current study

There is a gap in our understanding of social–emotional insight in autism, but evidence from non-autistic children shows that non-autistic girls demonstrate greater insight than non-autistic boys. We hypothesize that this difference between non-autistic girls and boys in social and emotional insight may be preserved in autistic girls and boys. In the current study, we investigate sex differences in the social and emotional insight of autistic youth and matched non-autistic peers by rating language produced during the interview sections of a commonly used diagnostic assessment, the Autism Diagnostic Observation Schedule-2nd edition (ADOS-2; [33]). Based on prior research, we test three hypotheses: First, the language produced by non-autistic children will be rated (by raters naïve to diagnosis) as containing greater social and emotional insight than language produced by autistic children, regardless of sex. Second, girls’ language will be rated as more insightful than boys’ language, regardless of diagnosis. Third, in an exploratory analysis, we looked at each diagnosis separately to examine whether sex differences in social and emotional insight remained significant. The overarching goal of this study was to further our understanding of social and emotional insight in autism, with a particular focus on potential sex differences that could impact the referral process, as well as clinical/diagnostic identification.

## Methods

### Participants

Sixty-four child and adolescent participants, thirty-two with autism (16 females) and thirty-two non-autistic (16 females) were selected from a pool of individuals who were seen at a large academic medical research center (Children’s Hospital of Philadelphia’s Center for Autism Research). Children participated in a larger series of studies that included autism diagnostic assessments, IQ testing (with a minimum score of 75 or higher to participate), and behavioral tasks. To match groups, participants with complete data (age, sex, race, ADOS-2 recordings, and IQ testing) were first selected from the larger pool. Participant sex was characterized using parent-reported assigned sex. The current study includes four groups based on sex and diagnosis: non-autistic girls, non-autistic boys, autistic girls, and autistic boys. Participants in these groups were individually matched on IQ (within 15 points) and chronological age (within 18 months). Girls and boys with autism were also individually matched for autism symptom severity (within 5 points on the SCQ scale). Participant characteristics and matching statistics are provided in Table 2 in Additional file 1.

Participants were recruited using a variety of methods, including public advertising, word-of-mouth, and re-recruiting from previous studies. Participants were excluded from the larger parent study if they had a known genetic syndrome, history of concussion or brain injury that impacted current functioning, history of medication use that caused permanent changes in motor behavior (e.g., amphetamines), gestational age below 34 weeks, or if English was not their primary language. Parents of participants provided written informed consent to participate in this study, which was overseen by the Children’s Hospital of Philadelphia.

### Measures

All participants completed the Autism Diagnostic Observation Schedule-2nd edition (ADOS-2 [33]), a clinician-administered assessment of the presence and severity of autism symptoms. Participants received Module 3 which requires fluent verbal skills, depending on their chronological age and the examiner’s clinical judgment. Verbal fluency was defined by an individual’s ability to demonstrate regular use of complex sentences, expressive language skills at or above a typical four-year-old level, produce a range of sentence types and grammatical forms, provide information beyond the immediate context, and use logical connections such as “but” and “because” [34]. Overall scores were calculated for the domains of Social Affect and Restricted and Repetitive Behavior [35]. Parents and caregivers completed the Social Communication Questionnaire (SCQ; [35]) to assess the presence of autism symptoms. Autism diagnoses were made by expert PhD-level clinicians using the clinical best estimate (CBE) approach [34]. The CBE method prioritizes DSM-5 criteria informed by family/medical history and an evaluation by an autism specialist.

All participants received a cognitive assessment. Clinicians administered either the Differential Ability Scales-2nd Edition (DAS-II; [36]), the Wechsler Abbreviated Scale of Intelligence-2nd Edition (WASI-II; [37]), the Stanford-Binet Intelligence Scales-5th Edition (SB5; [38]), or the Wechsler Intelligence Scale for Children-5th Edition (WISC-V; [39]), according to the protocol of the larger study from which the current sample was drawn. To allow for comparison across these assessments, scores were standardized and reduced to an overall cognitive estimate (Full Scale IQ), as well as Verbal IQ and Nonverbal IQ sub-scores by an expert licensed neuropsychologist, refer to Table 1.

**Table 1:** Demographic and clinical characteristics of participants (means, standard deviations, and ranges)

	Autistic (*N* = 32)		Non-Autistic (*N* = 32)		Effects		
Sex ratio	16f, 16 m (50% Male)		16f, 16 m (50% Male)		χ^2^ = N/A, *p* = N/A		
Race	Black or African-American: 3.1% (*n* = 1) White/Caucasian: 87.5% (*n* = 28) Asian or Pacific Islander: 3.1% (*n* = 1) Multiracial: 6.3% (*n* = 2) Other: 0% (*n* = 0)		Black or African-American: 37.5% (*n* = 12) White/Caucasian: 37.5% (*n* = 12) Asian or Pacific Islander: 6.3% (*n* = 2) Multiracial: 15.6% (*n* = 5) Other: 3.1% (*n* = 1)		χ^2^ = 18.32, *p* = .001		
Maternal Education	High school or less: 3.1% (*n* = 1) Bachelor’s or less: 46.9% (*n* = 15) Graduate degree: 28.1% (*n* = 9) Not reported: 21.9% (*n* = 7)		High school or less: 9.4% (*n* = 3) Bachelor’s or less: 28.1% (*n* = 9) Graduate degree: 34.4% (*n* = 11) Not reported: 28.1% (*n* = 9)		χ^2^ = 10.17, *p* = .118		

	Girls	Boys	Girls	Boys	Sex	Dx	Sex in Autism
Age (years)	Mean (SD) 10.46 (1.53) Range 8.67–13.25	Mean (SD) 9.60 (1.69) Range 7.42–12.50	Mean (SD) 9.99 (2.12) Range 6.75–13.60	Mean (SD) 9.88 (2.08) Range 7.01–12.75	*p* = .296 *F*(1.62) = 1.11	*p* = .841 *F*(1,62) = .04	*p* = .139 *F*(1,30) = 2.31
Full-Scale IQ	Mean (SD) 109.94 (9.97) Range 92.00–131.00	Mean (SD) 107.31 (11.58) Range 92.00–128.00	Mean (SD) 105.69 (15.18) Range 84.00–134.00	Mean (SD) 109.19 (12.94) Range 86.00–129.00	*p* = .889 *F*(1.62) = .02	*p* = .704 *F*(1,62) = .15	*p* = .497 *F*(1,30) = .47
Verbal IQ	Mean (SD) 109.75 (12.80) Range 87.00–134.00	Mean (SD) 108.44 (12.56) Range 83.00–130.00	Mean (SD) 107.75 (12.80) Range 84.00–129.00	Mean (SD) 108.88 (14.02) Range 86.00–130.00	*p* = .977 *F*(1,62) = .001	*p* = .806 *F*(1.62) = .06	*p* = .765 *F*(1,30) = .09
Nonverbal IQ	Mean (SD) 109.63 (10.87) Range 90.00–133.00	Mean (SD) 107.69 (12.25) Range 87.00–131.00	Mean (SD) 102.19 (15.52) Range 81.00–132.00	Mean (SD) 108.44 (12.69) Range 89.00–130.00	*p* = .510 *F*(1,62) = .44	*p* = .306 *F*(1,62) = 1.07	*p* = .639 *F*(1,30) = .22
ADOS-2 Total CSS	Mean (SD) 5.75 (2.27) Range 2.00–10.00	Mean (SD) 7.00 (2.50) Range 3.00–10.00	Mean (SD) 1.38 (.89) Range 1.00–4.00	Mean (SD) 1.31 (.60) Range 1.00–3.00	*p* = .448 *F*(1,62) = .58	*p* < .001 *F*(1,62) = 125.11	*p* = .149 *F*(1.30) = 2.19
ADOS-2 SA CSS	Mean (SD) 5.81 (2.14) Range 3.00–10.00	Mean (SD) 6.94 (2.38) Range 3.00–10.00	Mean (SD) 1.81 (1.27) Range 1.00–5.00	Mean (SD) 1.81 (.91) Range 1.00–3.00	*p* = .444 *F*(1,62) = .59	*p* < .001 *F*(1,62) = 103.06	*p* = .170 *F*(1,30) = 1.98
ADOS-2 RRB CSS	Mean (SD) 6.44 (2.66) Range 1.00–10.00	Mean (SD) 6.69 (2.60) Range 1.00–8.00	Mean (SD) 1.87 (1.92) Range 1.00–7.00	Mean (SD) 2.38 (2.16) Range 1.00–7.00	*p* = .644 *F*(1,62) = .22	*p* < .001 *F*(1,62) = 58.25	*p* = .790 *F*(1,30) = .07
SCQ Total	Mean (SD) 19.56 (6.85) Range 8.00–31.00	Mean (SD) 18.69 (6.34) Range 9.00–30.00	Mean (SD) 1.75 (2.86) Range 0.00–10.00	Mean (SD) 3.31 (3.53) Range 0.00–14.00	*p* = .890 *F*(1,62) = .02	*p* < .001 *F*(1,62) = 166.20	*p* = .710 *F*(1,30) = .1

Race was missing for one of the non-autistic participants. Maternal education was missing for seven of the autistic participants and for nine of the non-autistic participants. CSS = ADOS-2 calibrated severity scores; SA CSS = social affect calibrated severity score; RBB CSS = repetitive behaviors/restricted interests calibrated severity score, SCQ = Social Communication Questionnaire. Chi-squared tests for diagnostic group differences in race ratio and maternal education. *p* values for the main effects of sex and diagnosis are shown above, as well as *p* values for sex differences specifically in the autistic group

### Language sample

Language data were drawn from the interview sections of research-reliable administrations of the ADOS-2 Module 3, previously recorded at the Center for Autism Research at the Children’s Hospital of Philadelphia. For the purpose of these analyses, language data from the following ADOS-2 sections were included: emotions, social difficulties and annoyance, friendships, relationships and marriage, and loneliness. These conversations provide a rich semi-structured language sample that includes a discussion of diverse social topics, and were used to measure children’s insight into emotions and relationships. Breaks were not included in analyses. Conversations were audio/video recorded using standard free-standing video cameras.

Audio recordings of each conversation were orthographically transcribed by reliable annotators who were unaware of the participants’ diagnostic status and study hypotheses. Annotators were undergraduate student research assistants, trained on a modified Quick Transcription protocol for XTrans software [40]; all were trained on segmenting and transcription, with a minimum 92% word-level reliability criteria that must be met consistently before beginning to transcribe ([13, 41] for a more detailed description of transcription process). Once a final transcript was created for each participant from the annotators, a graduate researcher removed features from the transcriptions including words or phrases relating to the sex, name, location, and other factors that could be identifying factors. These words or phrases were replaced with non-sex-specific terms or removed. Any removed or rephrased words did not change the meaning of the verbal exchange. The transcriptions were then modeled after a script such that the person speaking would be identified followed by what they said. Finally, transcriptions were uploaded into a Qualtrics online experimental format for the purpose of this study.

### Rating procedure

Two undergraduate students majoring in linguistics at a small eastern liberal arts college served as raters for this study. The students underwent a training process that lasted approximately six weeks. During training, the raters were given transcripts each week, which they would read and analyze, and then rate each transcription of a participant’s conversation using scales designed to capture insight (see *Insight Scales*). Raters were not informed of participants’ diagnosis, sex, or age. A total of 30 transcripts were used to establish initial reliability between raters on the insight measures. During the training process, consistent reliability was established between the two raters on each of the four insight scales and tested using an intraclass correlation coefficient [42]. After training was complete, the raters then began the process of rating the transcripts. Throughout the study, the raters were unaware of study hypotheses related to sex differences or autism, in order to eliminate potential bias. Approximately 44% (28/64) of study transcripts were double-scored by both raters, and the remaining were single-scored. Transcripts that were doubled scored had reliability ratings of moderate-to-high (see *Results* section for details).

### Insight scales

Four different insight scales were used to rate each transcript. Together, these scales assessed the participants’ social and emotional insight based on the language they produced during the interview sections of the ADOS-2 (refer to Table 2). Insight scales were drawn from previous studies measuring insight in non-autistic children, with modifications to improve clarity and to make them more suitable for the age range of our study sample (see Additional file 1 section for details). With regards to the adaptations that were made, they involved changing the language to be more age-appropriate for our sample (age 7–14). The five levels of emotional awareness in children were worded for assessment on young children; the wording was modified to fit an older age range. The other three scales (social cognition and object relation, emotional investment in relationships, and understanding of social causality scale) were created for adult populations, given the current sample the scale was modified to be phrased for youth and adolescents.

**Table 2:** Average scores on insight scales by sex and diagnosis (means, standard deviation, and range)

Insight scales	Autism (*N* = 32)		Non-autism (*N* = 32)		Effects		
	Girls	Boys	Girls	Boys	Sex	Dx	Sex in autism
Five levels of emotional awareness	3.19 (.201) 2.00–4.00	2.75 (.201) 1.00–4.00	3.44 (.201) 1.50–5.00	2.94 (.201) 2.00–4.00	*p* = .023* *η*^2^ = .08	*p* = .281 *η*^2^ = .07	*p* = .120
Emotional investment in relationships	3.16 (.243) 2.00–4.00	2.75 (.243) 1.00–4.00	3.84 (.243) 2.00–5.50	3.13 (.243) 1.50–5.00	*p* = .058 *η*^2^ = .06	*p* = .013* *η*^2^ = .10	*p* = .188
Social cognition and object relations	3.31 (.257) 2.00–4.00	2.60 (.257) 1.00–4.00	4.03 (.257) 2.00–6.00	3.03 (.257) 1.50–5.50	*p* = .001* *η*^2^ = .16	*p* = .028* *η*^2^ = .08	*p* = .020*
Understanding of social causality	3.44 (.253) 2.00–4.50	2.88 (.253) 1.00–4.00	4.16 (.253) 2.00–6.50	3.22 (.253) 1.00–5.00	*p* = .004* *η*^2^ = .13	*p* = .040* *η*^2^ = .07	*p* = .041*

#### Five levels of emotional awareness in children scale

This scale indexes participants’ ability to verbally describe different emotions and was rated on a 5-point scale (1 being minimal to no ability to 5 having the greatest ability) [43]. This scale was used to rate participants’ responses to direct questions about emotions, e.g., how they would describe those emotions, how their body feels when they are experiencing a specific emotion, and to recall a time when they experienced that emotion (see Additional file 1: Appendix 1-A). A moderate degree of reliability was found between raters on the Five Levels of Emotional Awareness in Children scale (ICC = 0.737, 95% CI [0.43–0.88], *F*(1,25) = 4.23, *p* < 0.001).

#### Social cognition and object relations scale

This scale was used to index participants’ understanding of their own and others’ thoughts and emotions using a seven-point scale (1 = minimum understanding of their own and others’ thoughts and emotions, 7 = a very deep understanding of their own and others’ emotions and thoughts) [44]. Participants’ direct responses to questions relating to emotions, relationships, and annoyances were included in this rating. This scale has a direct link with a person’s ability to take another person’s perspective, and measures “perspective-taking” ability in a quantifiable way (see Additional file 1: Appendix 1-B). A high degree of reliability was found between raters on the Social Cognition and Object Relations scale (ICC = .801, 95% CI [0.49, 0.92], *F*(1,25) = 6.28, *p* < 0.001).

#### Emotional investment in relationship scale

This scale was used to index the participants’ level of insight into their role in various relationships, as well as their emotional investment in those relationships, using a seven-point scale (ranging from 1 = only focusing on themselves in a relationship, to 7 = the participant develops deep committed relationships with mutual understanding) [44]. The rating was based on the highest-level relationship expressed by the participant, due to the nature of different relationships being deeper or shallower depending on context (see Additional file 1: Appendix 1-C). A moderate degree of reliability was found between raters on the emotional investment in relationships scale (ICC = 0.693, 95% CI [0.35, 0.86], *F*(1,25) = 3.30, *p* = 0.001).

#### Understanding of social causality scale

This scale was used to index participants’ ability to recall past events/experiences, including emotions that they felt during that event/experience, and how their behaviors impacted others involved, on a seven-point scale (1 = limited ability to recall past events/experiences, to 7 = the individual recalls very coherent and precise past events/experiences in great detail, not only of themselves but others present as well) [44]. This scale combined multiple areas of interest such as emotions, understanding of their own behaviors, recall of experiences, and different relationships (see Additional file 1: Appendix 1-D). A high degree of reliability was found between raters on the understanding of social causality scale (ICC = 0.760, 95% CI [0.39, 0.90], *F*(1,25) = 5.31, *p* < 0.001).

### Statistical approach

To test the core experimental hypotheses of the study, four 2 × 2 general mixed model ANOVAs were conducted; one for each of the four insight scales (independent variables). Preliminary analyses revealed no significant interactions between diagnosis and sex (all *p* > 0.40), so final analyses were conducted with simple main effects only. Covariates of age and IQ were tested and no significant differences between groups were found. Groups were matched on age, IQ and SCQ scores for the autism group. The main effects of diagnosis and sex are reported separately. Additionally, ANOVAs were conducted between the autistic girls and boys to test whether sex differences observed in the larger group were present in the autistic group alone. All statistical analyses were conducted using IBM SPSS (Version 29.0).

## Results

### Effects of diagnosis

**Fig. 1:**
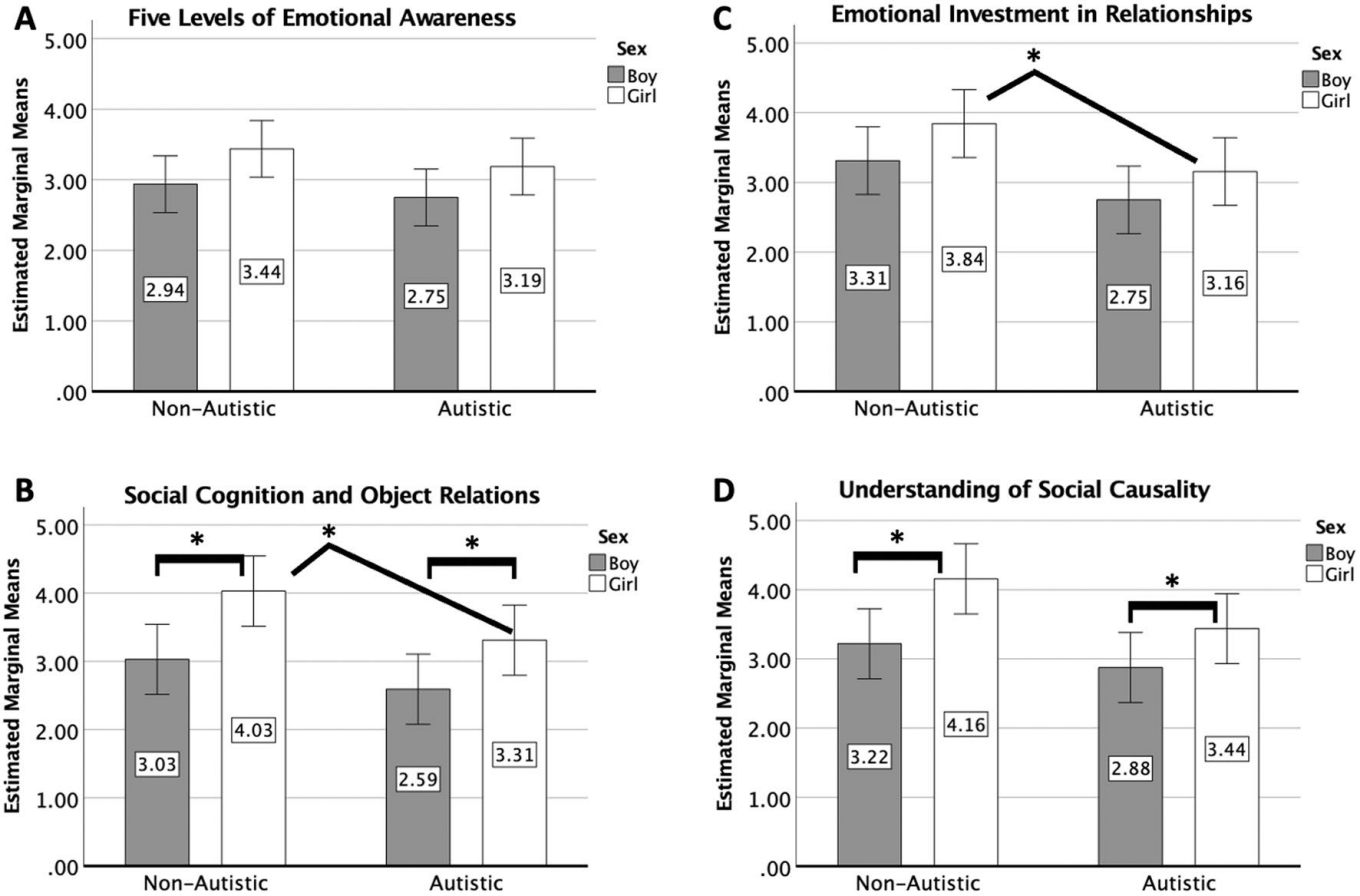
Estimated marginal means of ratings on the four scales by sex and diagnosis. Presented above are data from each of the four insight scales, sex means (boy—gray; and girl—white) and diagnosis means (no autism diagnosis and autism diagnosis) are shown. For the figure above, *p < .05, **p < .01, and ***p < .005. **A** Results from an ANOVA show that girls were significantly rated higher on insight measured by the five levels of emotional awareness scale, *p* = .023. Differences between diagnosis, *p* = *.281* and an interaction between sex and diagnosis, *p* = .877, were not significant. **B** Results from an ANOVA show that non-autistic individuals were rated significantly higher than individuals with an autism diagnosis, *p* = .013. Differences based on sex, *p* = .058, and an interaction between sex and diagnosis, *p* = .798, were not significant. Additionally, differences in girls were found *p* = .046. **C** Results from an ANOVA show that non-autistic individuals were rated significantly higher than individuals with an autism diagnosis, *p* = .028, and sex, *p* = .001. In the non-autistic group, girls were rated significantly higher than boys, *p* = .025. Similarly, in the autism diagnosis group, girls were rated significantly higher than boys *p* = .020. Differences in an interaction between sex and diagnosis, *p* = .587, were not significant. Additionally, differences in girls were found *p* = .044. **D** Results from an ANOVA show that non-autistic individuals were rated significantly higher than individuals with an autism diagnosis, *p* = .040, and sex, *p* = .004. In the non-autistic group, girls were rated significantly higher than boys, *p* = .038. Similarly, in the autism diagnosis group, girls were rated significantly higher than boys *p* = .041. Differences in an interaction between sex and diagnosis, *p* = .462, were not significant

Results revealed significant diagnostic group differences on these three following scales: social cognition and object relations (*F* = (1,63) = 5.05, *p* = 0.028, partial *η*^2^ = 0.08 with an observed power of 0.60), the non-autism group was rated higher (*M* = 3.53, SD = 1.28) than the autism group (*M* = 2.95, SD = 0.89). Emotional investment in relationships scale (*F* = (1,63) = 6.62, *p* = 0.013, partial *η*^2^ = 0.10 with an observed power of 0.71), the non-autism group was rated higher (*M* = 3.58, SD = 1.10) than the autism group (*M* = 2.95, SD = 0.86). The understanding of social causality scale (*F* = (1,63) = 4.40, *p* = 0.040, partial *η*^2^ = 0.07 with an observed power of 0.54); again, the non-autism group scored higher (*M* = 3.69, SD = 1.30) than the autism group (*M* = 3.15, SD = 0.79). There were no significant diagnostic group effects on the five levels of emotional awareness in children scale (*F* = (1,63) = 1.18, *p* = 0.281, partial *η*^2^ = 0.02 with an observed power of 0.19), non-autism group (*M* = 3.19, SD = 0.79) and autism group (*M* = 2.97, SD = 0.79). See Fig. 1 and Table 2.

### Effects of sex

In the overall sample, there were significant differences found between girl and boy participants on the following three scales: five levels of emotional awareness in children (*F* = (1,63) = 5.43, *p* = 0.023, partial *η*^2^ = 0.08 with an observed power of 0.63), girls scored higher (*M* = 3.31, SD = 0.81) than boys (*M* = 2.84, SD = 0.79). Social cognition and object relations scale (*F* = (1,63) = 0.11.162, *p* = 0.001, partial *η*^2^ = 0.16 with an observed power of 0.91), girls scored higher (*M* = 3.67, SD = 1.02) than boys (*M* = 2.80, SD = 1.09). The understanding of social causality (*F* = (1,63) = 0.8.78, *p* = 0.004, partial *η*^2^ = 0.13 with an observed power of 0.83), girls scored higher (*M* = 3.80, SD = 1.14) than boys (*M* = 3.05, SD = 0.92). The were no significant differences found between sexes on the emotional investment in relationships scale (*F* = (1,63) = 3.72, *p* = 0.058, partial *η*^2^ = 0.06 with an observed power of 0.48) girls (*M* = 3.50, SD = 0.98) and boys (*M* = 3.00, SD = 1.03). See Fig. 1 and Table 2.

### Effects of sex in the autism group only

An ANOVA testing the effect of sex on social–emotional insight in the autism group alone revealed significant differences on two of the four scales: social cognition and object relations (*F*(1,31) = 6.03, *p* = 0.020), girls scored higher (*M* = 3.31, SD = 0.63) than boys (*M* = 2.60, SD = 0.97) and understanding of social causality scale (*F* = (1,31) = 4.55, *p* = 0.041), girls scored higher (*M* = 3.44, SD = 0.68) than boys (*M* = 2.88, SD = 0.81). The results of the ANOVA between sexes on individuals with autism indicated no significant results on two of the four scales. Five levels of emotional awareness in children (*F* = (1,31) = 2.56, *p* = 0.120), girls (*M* = 3.19, SD = 0.63) and boys (*M* = 2.75, SD = 0.89) and emotional investment in relationships scale (*F* = (1,31) = 1.82, *p* = 0.188), girls (*M* = 3.15, SD = 0.81) and boys (*M* = 2.75, SD = 0.89). See Fig. 1 and Table 2.

## Discussion

The goal of this study was to assess differences in social and emotional insight in youth with autism compared with matched non-autism controls, with a particular focus on potential variation by sex. Transcriptions of selected sections of the ADOS-2 (Module 3) interview portion were rated by trained undergraduate students who were experimentally masked, using the following scales: Five Levels of Emotional Awareness, Social Cognition and Object Relations, Emotional Investment in Relationships, and Understanding of Social Causality. Results revealed that levels of social and emotional insight differ between autistic and non-autistic participants and, between boys and girls. Specifically, autistic children’s insight was rated lower than that of non-autistic peers on 3 of the 4 scales. Additionally, girls exhibited higher levels of insight compared with boys, regardless of diagnosis, for 3 of the 4 scales. Finally, girls with autism exhibited higher levels of insight than boys with autism on 2 of the 4 scales.

The hypothesis that decreased levels of social and emotional insight would be present in individuals with autism was supported by our finding of lower mean ratings on all four of the insight scales for the autism group, with significantly lower mean rating scores for three of the four scales (social cognition and object relation, emotional investment in relationships, understanding of social causality scales). Furthermore, there was no interaction of diagnosis with sex, indicating that participants with autism exhibited lower levels of insight regardless of their sex. These findings are consistent with previous research indicating that individuals with autism exhibit difficulties with different social concepts, including emotional understanding and understanding of different interpersonal relationships [9, 45]. To date, the vast majority of research has focused on difficulties that individuals with autism exhibit in using social skills, with much less research designed to determine whether there are differences in their understanding of social and emotional topics. To our knowledge, this is the first study to analyze insight and understanding of these social and emotional topics in individuals with autism versus non-autistic individuals.

With regards to sex differences, we report mean insight scores which were higher for girls versus boys on all 4 scales, with significant differences observed for the five levels of emotional awareness in children, social cognition and object relations scale, and understanding of social causality scale. These results support our second hypothesis that girls, regardless of diagnosis group, would be rated higher on levels of social and emotional insight when compared with boys of similar age, intellectual abilities, and autism symptom scores. These findings are consistent with previous research which has found that non-autistic girls exhibit greater social understanding and insight compared to boy peers [9]. However, to our knowledge, the current study is the first to analyze sex differences in insight levels across samples of youth both with and without an autism diagnosis. The current findings, therefore, replicate previous findings indicating higher levels of social and emotional insight in non-autistic girls versus boys and further extend these findings by providing direct evidence that this same pattern may be present in girls versus boys with autism. Furthermore, the use of 4 different social and emotional rating scales in the current study along with the absence of a diagnosis by sex interaction suggests that sex differences in social and emotional insight may be very similar in the autism and non-autism populations.

While there was no significant interaction between sex and diagnosis on any of the social or emotional rating scales, we did conduct follow-up statistical tests examining sex differences separately for the autism and non-autism groups, in order to confirm whether or not each of the two groups exhibited the sex differences on their own. The results of these analyses indicated that girls scored significantly higher than boys in both the autism and non-autism groups for the social cognition scale and the understanding of social causality scale, but not for either of the emotional insight scales. These findings suggest that sex differences are most pronounced and/or most consistent in the domain of social insight than in the domain of emotional insight, for both groups. Thus, enhanced social cognitive insight and enhanced insight into social causality for girls versus boys appear to be a consistent population-level sex difference that is preserved in autism, despite the fact that girls with autism exhibit impaired social behavior and social insight relative to non-autistic girls.

The current results indicate that girls with autism demonstrated higher levels of social insight when compared to boys with autism of similar chronological age, intellectual ability, and autism symptom scores. These findings support the hypothesis that girls with autism present with elevated levels of social insight than boys with autism, which was directly supported by girls with autism being rated as significantly more insightful on both the social cognition and object relation scale and the understanding of social causality scale. This pattern of results is consistent with previous research suggesting that girls with autism may have a greater understanding of social topics when compared with boys with autism [9]. However, while girls with autism were rated higher in social insight levels compared with boys with autism, the girls with autism still exhibited lower levels of social insight when compared with non-autistic girls. These findings suggest that, even while girls exhibit an elevated level of insight when compared to boys, an autism diagnosis is still associated with reduced levels of insight on social topics even in girls. Higher levels of social insight while still presenting with reduced social insight compared with non-autistic girls may also help explain why autistic girls tend to have reduced symptoms relative to autistic boys.

### Potential explanations

One potential explanation for the current finding of sex differences in girls versus boys relates to both the quality and quantity of social experiences between boys and girls. When examining peer interactions between girls and boys, girls have been found to heavily rely on social and communicative interaction when forming and maintaining relationships [31]. In previous studies, girls were found to typically put themselves in more situations that focused on socializing with other peers and activities that were centered around talking and being around others without structure (e.g., [30]). This differential experience, in turn, exposes girls with and without autism to more opportunities to learn about and practice different social situations. These sex-specific stereotypes present in today’s society may play a critical role in the sex differences in social and emotional insight in the current study and others.

Along with the potential for direct impacts on social and emotional insight, sex-specific social demands and sex-specific social experience may also impact social and emotional functioning, motivation, and engagement in other ways. When mothers talked to their children, there is typically a difference in the topics discussed between genders [46]. Talking to their sons, the conversations are usually more about learning and instruction, while talking to their daughters the conversations focus more on social interaction and emotions. The topics of conversations have then been observed to be what the child focuses on during free time, either instruction-based play or social play [31].

In addition, girls who face rejection and bullying are often met with more mental harassment [44]. In contrast to this, boys who are rejected are often met with more physical bullying and harassment, yet girls are more impacted by the mental bullying and harassment they experience [47]. Mental bullying refers to name-calling, group exclusion, and talking behind someone’s back, which girls experience more frequently [47]. Therefore, girls and boys, including autistic girls and boys, experience different risks if and when they exhibit reduced social and/or emotional insight. Besides the potential to lead to anxiety, depression, and social isolation, this type of bullying may also lead individuals to learn particular social behaviors and characteristics in an effort to better fit into societal norms and to avoid bullying.

As previously discussed, autism throughout its history has been primarily studied and diagnosed using male-dominated samples [48]. At the same time, most early-onset developmental disorders (i.e., those identifiable within the first 6 years of life) are more commonly diagnosed in boys and the populations are, therefore, heavily male-dominated [5]. Therefore, one possible explanation for the current study findings and their relationship to the identification and diagnosis of girls with autism is that girls simply are not affected by autism at the same rate as boys, and that when girls are affected by autism one of their characteristics is that they exhibit more intact insight into social relationships, reflecting a more mild form of internal social cognitive difficulty. However, several other potential explanations might suggest that current clinical and diagnostic tools and practices might need to be reconsidered and modified.

Another potential relationship between the current study’s findings and current efforts to identify and diagnosis girls with autism relates to the potential for sex differences in camouflaging. As described earlier, camouflaging is the act of modifying behaviors to fit situational contexts [41]. The finding of greater social insights in girls versus boys with autism in the current study opens up the possibility that girls with autism may, on average, be more capable than boys with autism when it comes to camouflaging their behavioral social interaction difficulties and hiding them from others. If this were the case, then girls with autism may be harder to identify and diagnose than boys with autism; yet, as highlighted previously by Parish-Morris, these girls may be experiencing internal symptoms such as negative emotionality as a result of their social experience and lack of support as an un-diagnosed or mis-diagnosed individual [49].

### Limitations

As with any study, the current study has some limitations that need to be addressed when continuing this type of work in the future. The first limitation is related to the age of our sample. The current study focused on children and adolescents; thus, we are not able to generalize whether the observed insight differences persist into adulthood or, instead, if age and/or experience increases or decreases the gaps in social and emotional insight. Another limitation of the current study is that the sample consisted of autistic youth without co-occurring language impairments or intellectual disabilities. Due to the sample, we cannot generalize the findings of sex differences in social and emotional insight to autistic individuals who have co-occurring language impairments or intellectual disabilities. The study results should be interpreted and generalized with consideration for the study sample, which is primarily white youth with average to high average cognitive abilities and mild to moderate autism symptoms as measured by the ADOS-2 and SCQ. Future research on this topic will need to use broader samples in order to produce a more clear understanding of how insight varies for all autistic individuals across the spectrum. The current study measured insight by transcribing and scoring participants’ verbal responses to questions related to social and emotional insights on a standardized clinical/diagnostic assessment. Measuring insight this way allows us to measure a participant’s outward expression of social and emotional insight through verbal language is an objective way to index internal states through self-report. However, this approach does not necessarily capture the complete level of insight present in the individual. Future research should attempt to expand upon the current study by developing measures that probe deeper into the potential for additional internal aspects of insight.

### Future directions

The results of the current study provide direct evidence for differences in levels of social and emotional insight between autistic girls and boys. These findings help to further the field’s understanding of unique differences potentially present in autistic girls versus boys. Currently, little is known about autistic girls and women due to females accounting for only a small percentage of the autistic population. The current findings, while promising, are one of only a relatively small number of studies on this topic and population. More research is needed to draw firmer conclusions and to develop more comprehensive theories related to girls and women with autism.

There are three future directions which we believe should be taken. The first is related to examining the potential for modifications of diagnostic tools and processes or creation of new tools and processes to better serve not currently diagnosed autistic girls. A majority of the current diagnostic tools in use today for autism diagnosis were standardized using primarily male populations with only 22% of female participants [50]. Recent research studies, including this study, are starting to demonstrate subtle but important differences in the characteristics and symptoms of autism in girls and women when compared to boys and men. With new information and ideas about autistic girls produced by the current study, it is clear that diagnostic tools and processes need to be examined in greater detail in order to assess if they need to be modified to address the likely differences in social and emotional insight between autistic boys and girls. For example, just as clinical/diagnostic training explicitly addresses the need for clinicians to consider the chronological and developmental age of each participant when evaluating and scoring patient responses to questions probing social and emotional insight, the results of the current research indicate that these trainings should also explicitly address the need for clinicians to consider the sex of the patient. Future research using larger samples of participants should also directly examine the impacts of sex and gender on ADOS-2 clinical/diagnostic insight-related and other scores given to girls versus boys, and women and men, as well as how these relate to Clinical Best Estimate diagnosis decision-making.

Second, there is a need to conduct studies that will help to determine how best to modify different interventions to better serve autistic girls and women. The results of this study present a difference in the insight into social and emotional topics in autistic girls and boys. In addition to these observed differences, the explanation of social motivation theory suggests that there are not only differences in understanding of social topics, but additionally in how this understanding leads to behaviors and attempts to form bonds based on these understandings [11, 51]. Future research should examine the real-world impacts of deficits and differences in social and emotional insight in girls versus boys, including how these deficits and differences impact their interactions, comfort, self-advocacy, and success in interactions with both same-sex and opposite-sex individuals and groups. For example, how autistic girls are impacted by having lower social and emotional insight than their non-autistic female peers, and how they are impacted by having similar social and emotional insight as their non-autistic male peers. The results of these studies could lead to intervention programming that is more tailored to, and more impacting for, autistic girls.

Finally, there is a need to conduct follow-up research in order to more fully characterize and understand the nature, depth, and breadth of social and emotional insight differences across autistic and non-autistic girls and boys, and women and men. For example, although the transcripts utilized in the current study, which were derived from a relatively short discussion relevant to social and emotional insights between a clinician and a patient, have provided the opportunity to examine and compare social and emotional insight in autistic and non-autistic girls and boys, the transcripts are insufficient for a qualitative or thematic analysis. Future research which involves developing and deploying more involved interview-based assessments of social and emotional insight in autistic and non-autistic girls and boys will be very valuable for ensuring that we come to a more complete understanding of the nature, extent, depth, and variability of sex differences in social and emotional insight.

## Conclusions

In summary, the findings of the current study provide evidence for differences in insight into social topics between girls and boys with and without autism. Specifically, youth with autism were found to exhibit lower levels of social and emotional insight than youth without autism. Furthermore, girls were found to exhibit higher levels of social cognitive and social causality insight than boys, regardless of diagnosis. To date, there has been minimal research examining differences in boys and girls with autism, in general, or into social insight of girls and boys with autism. The current findings are consistent with previous research demonstrating that non-autistic girls exhibit higher levels of social insight when compared to non-autistic boys, and further extend this finding with evidence of this same effect in a sample of children diagnosed with autism.

The findings of the current study have direct implications for understanding the characteristics, experiences, and abilities of girls with autism. For example, the fact that girls with autism have higher insight into social cognition and social causality than boys with autism but lower insight into social cognition and social causality than non-autistic girls suggests that interventions and supports need to consider the role and relationships of girl-specific social interactions and experiences to the experience of autism in girls. Similarly, clinicians should be made explicitly aware that there are sex differences in social insight in girls and boys with and without autism, which may need to be taken into account when assessing girls for autism using existing standardized and non-standardized clinical-diagnostic procedures. For example, just as clinicians take patient developmental and chronological age into consideration when setting their expectations for administering and scoring the Autism Diagnostic Observation Schedule (ADOS) for topics including social and emotional insight, clinicians may also need to consider patient sex as a factor when administering and scoring the social and emotional insight sections of the ADOS-2. The current findings also have implications for furthering our understanding of the expressions and experiences of autism more generally. For example, by further elucidating the autistic girl phenotype in the current study, we can begin to imagine and plan studies that will help us to understand the full diversity of developmental processes, mechanisms, and internal and external cognitive and emotional experiences of people with autism, and to develop and test theoretical models of autism.

## Supplementary Information

**Additional file 1.** Provided in the file are the four insight scales used in the current study. The scalesare: Five Levels of Emotional Insight, Emotional Investment in Relationships, Social Cognition and ObjectRelationships, and Understanding of Social Causality.

## Data Availability

The datasets generated and/or analyzed during the current study are not publicly available due to privacy concerns for minors with disabilities.
