# Supplementary material for: Sex differences in social and emotional insight in youth with and without autism
Source: Mol Autism. 2023 Mar 4;14:10. doi: 10.1186/s13229-023-00541-w (PMC9985847; doi:10.1186/s13229-023-00541-w)
Supplement: Supplementary file 1 — Additional file 1. Provided in the file are the four insight scales used in the current study. The scalesare: Five Levels of Emotional Insight, Emotional Investment in Relationships, Social Cognition and ObjectRelationships, and Understanding of Social Causality. [file 13229_2023_541_MOESM1_ESM.docx]

**Supplemental Materials**

**Appendix 1-A**

Bajar et al., 2005

Five Levels of Emotional Awareness with Examples Scale

| Level | Ability to Describe Emotion | Example of Response |
| --- | --- | --- |
| 1 | No response | No response, Umm, Uh or I don’t know. |
| 2 | Bodily Sensation | When I go to the doctors, my stomach gets upset. I don’t know how the doctor feels. |
| 3 | One-dimensional emotion | Both the doctor and I would be nervous! |
| 4 | Differentiated Emotions | I would feel scared and worried. The doctor would feel calm and happy. |
| 5 | More complex and Differentiated States | I would feel worried because I might be sick but also excited because the doctor can help me feel better. The doctor would be happy and hopeful. |

**Appendix 1-B**

Stein et al., 2011

Social Cognition and Object Relations Scale

1 = Egocentric, thinks only of themselves in thoughts and feelings. Is unable identify that others have thoughts and emotions that are different than their own. Person only thinks of themselves. When asked to describe how others may feel in situations, they are unable to, or only describe how they feel and how that should be how the other person feels.

2 = Ability to describe basic emotions and thoughts of oneself. Able to identify that others have different thoughts and feelings from their own. Person is able to describe basic level emotions (happy, sad, mad, etc.) but has difficulty with higher level and more complex emotions. The person is now also able to identify that their emotions and thoughts are different from other people.

3 = Ability to minimally describe and identify basic thoughts and emotions of others. Typically classifies others’ thoughts as either good or bad. Person is now able to not only identify that others have emotions, but to identify what those emotions are (from facial cues, verbal communication, etc.). They are able to do so with basic level emotions. The person is also able to classify if those thoughts of other people are typically good or bad.

4 = Ability to describe others’ thoughts and emotions is almost average. Person is able to determine if a person’s characteristics are good or bad. Ability to describe others is average (can describe basic emotions: happy, sad, mad) without being able to fully explain the potential reasoning behind these emotions. Person determines if another person’s characteristics are good or bad based off of seen/ experience behaviors. (Ex. A person says their friend is good because when they are sad their friend tells jokes to cheer them up.)

5 = Representations of the self and others are typical and conventional. Person is able to combine both characteristics of good and bad into their selves and into others. This person is also aware that they have an impact on others and are able to identify said impact. (Person has a typical understanding of themselves and others. There appears to be no deficits in understanding others or themselves, but there is room for improvement, to understand on a deeper level. At this level the person understands that a person can have both good and bad characteristics but that does not necessarily make them a “good or bad” person. If person can identify the impact their emotions/ behaviors have on others 50% of the time they are asked they qualify for this level. Example: When the interviewer says something along the lines of “why do you think people might bully you and how can you change that?)

6 = Person has a great ability to determine representation of their selves and others. Person is able to place their and others’ thoughts into categories deeper than just good or bad. The person is aware of the impact they have on others and attempts to control these impacts. (Representation of themselves and others is just how they view themselves/others based on past experiences and how that person reacted, and how that would be seen in society. The categories going beyond just good or bad would be similar to level 4, but these would be more ideas/ concepts that are not physically seen. In the example earlier the participant said that the person was good because they told them jokes to cheer them up. In this level the person might say something along the lines of when I was sad my friend supported me and helped me to feel better. They were able to describe good characteristics without describing something that has to be visually seen. Lastly, for attempting to control impacts they have on others this would also play into the bullying question and would look something along the lines of “I know that a lot of the time I get picked on when I talk in a silly voice, so when I am around bullies, I don’t talk in a silly voice.”

7 = This person has a great knowledge of insight of oneself and a great understanding of others. Person can understand complex emotions in themselves and others around them. (This level is saved for very high-level insight. This is not typically seen in children and adolescents. This rating would only be given if the participant demonstrates an extremely highly level of insight. There are participants who seem highly insightful in certain areas, but this would be in a majority or all of the insight (emotions, relationships) discussed. And how that not only applies to themselves but others as well.

**Appendix 1-C**

Stein et al., 2011

Emotional Investment in Relationship Scale

1 = Person focuses primarily on their own needs in a relationship. This person also has very few relationships if any.

2 = Person places primary focus on themselves in the relationship but understands that a relationship is a based on two people. Very few relationships. In this level the person is primarily focused on themselves in the relationship. They actively understand that relationships (this can be friend, romantic, etc.) consist of two people, but they do not put in much if any effort to make the relationship two way. Person has very few relationships because of this misconception. The difference between level 2 and level 3 is that level 2 the person can understand that a relationship is built off of two people (passive). Level 3 they actively put work into that relationship (active).

3 = Person has shallow friendships, begins to place focus on both people in the relationship but primary focus is still the individual. Person has several relationships. In this level the person is beginning to shift their focus from solely on their selves to more of a two-way street relationship. While they understand and are actively putting effort into relationships it is not 50/50 from them and the other person. It would look something like them putting 25% of the effort into the relationship and the other person putting in 75%. The person is only able to describe one consistent relationship, other relationships seem either very shallow or inconsistent. (Ex. Participant has really steady and consistent relationship with sibling, but friends seem very surface level and not consistent).

4 = Person is able to shift the primary focus off of themselves and to the relationship as a whole. Person has consistent relationships. The person is able to realize that relationships require both people to put in equal effort so the relationship is more 50/50 at this level. The person realizes that the focus of the relationship should be on both parties involved, equally. During this level the person may start to referring to the relationship by saying “we” or “us” on occasions (signifying the 50/50 effort in the relationship). The person is able to describe more than one relationship which is consistent

5 = Person demonstrates conventional sentiments of friendship such as caring, loving and empathetic. Person has several close and consistent relationships.

6 = Person has committed relationships built on trust, mutual interests and reciprocal respect. Person has several committed relationships.

7 = Person tends to have very deep and committed relationships built on mutual sharing, emotional intimacy, interdependence, respect and appreciation of the other person. Person has several intimate and deep relationships that are consistent and dependable.

**Appendix 1-D**

Stein et al., 2011

Understanding of Social Causality Scale

1 = The ability to describe interpersonal experiences are limited in awareness in coherence. These experiences are often confused, distorted, lacking in numbers and examples and difficult to follow.

2 = The person’s ability to recall and describe interpersonal experiences are still limited in awareness and coherence. These experiences are often one sided, lacking of emotions, confusing to follow and consist of few examples. The person’s ability to recall and retell emotions is very one sided and focuses more on themselves and the role they played in the even as opposed to telling the story from both parties. The retelling of the story is often confusing to follow and when asked to recall a situation their ability to do so is limited with few examples. (Ex. “Tell me a time when you were happy” and the participant is only able to recall one or two times they were happy and they really have to take time to think about it).

3 = The individual understands people in a relatively simple way but sensible way. The person has the capability to describe interpersonal experiences and events in a way that makes sense but is lacking in some details that create gaps in the recalling of the event/experience. The participant is able to recall and retell experiences in a simple way, describe the event for more of the event and less about emotions felt and consequences during that experience. Emotions/consequences are mentioned in at least 50% of the retellings but not all of them. There are some gaps in the story telling at least 50% of the time.

4 = The person understands people more than just on the surface level emotions and experiences, they are beginning to develop a greater understanding of themselves and others. They are able to describe their experiences and past events with minimal gaps and disparities. The participant is able to recall and retell events by using surface level emotions (happy, sad, surprised, mad). They are not only telling what happened in the situation/experience but how that made them feel. Experiences are told with detail leaving minimal gaps and storytelling is consistent at least 50% of the time.

5 = The individual provides straightforward and easy to understand narrative accounts of their interpersonal experiences or events in which other’s actions result from the what they experience or how they interpret situations. Person is able to recall and retell events in their personal life with detail including different emotions that are deeper than surface level. The person is also able to identify that their actions and behaviors have an impact on their consequences and how others around them feel.

6 = This person is able to give clear and concise descriptions of their interpersonal experiences and events. They are also able to adequately understand the impact of their behaviors and actions on themselves and others. Person is able to recall and retell events in their personal life with great detail, using examples, feelings and consequences not only to themselves but to others as well. The person is able to identify that their actions and behaviors have an impact on themselves and others and can understand that they are able to change their behaviors to change these feelings/consequences to others.

7 = The individual is able to provide very coherent and precise accounts of their interpersonal experiences and events. This person understands other people, the impact of their behavior on themselves and others very well. Person is able to recall and retell events in great detail, with a focus on not only self but all parties revolved in the experience or event. The person is able to not only identify the role their actions and behaviors have on others, but actively modifies their behaviors to positively impact their relationship with others.
